# A case of herpes zoster following the first dose of benralizumab 

**DOI:** 10.5414/ALX400616

**Published:** 2026-04-17

**Authors:** Kurtuluş Aksu, Onur Telli, Melis Yağdıran, Fatma Dindar Çelik, Hatice Çelik Tuğlu, Özge Göktürk

**Affiliations:** Division of Immunology and Allergy, Department of Chest Diseases, Ankara Atatürk Sanatoryum Training and Research Hospital, University of Health Sciences, Ankara, Turkiye

**Keywords:** severe eosinophilic asthma, benralizumab, herpes zoster

## Abstract

Introduction: Biological agents used in the treatment of rheumatic diseases and malignancies cause an increase in herpes zoster susceptibility. This may also be the case for monoclonal antibody treatments used in asthma based on recent pharmacovigilance data. Case report: Herpetic lesions occurred on the anterior chest wall of a 43-year-old patient who was initiated on benralizumab treatment for severe eosinophilic asthma. The patient received diagnosis of herpes zoster and showed significant clinical improvement with oral valacyclovir treatment. Since it was not certain whether the herpes eruption was directly related to benralizumab treatment, benralizumab treatment was continued at the patient’s request and with the approval of the dermatology department. The patient did not experience recurrent herpes eruptions or any other clinical problems with the repeated doses given.

## Introduction 

Herpes zoster (HZ) develops as a result of the reactivation of latent *Varicella zoster virus* [1]. Many biological agents used in the treatment of rheumatic diseases and malignancies have been reported to increase the risk of HZ [2]. It is thought that biologics used in the treatment of asthma may also be associated with HZ. We present a case of HZ that developed after the first dose of benralizumab, a biological agent used in the treatment of severe eosinophilic asthma. 

## Case report 

**Figure 1:**
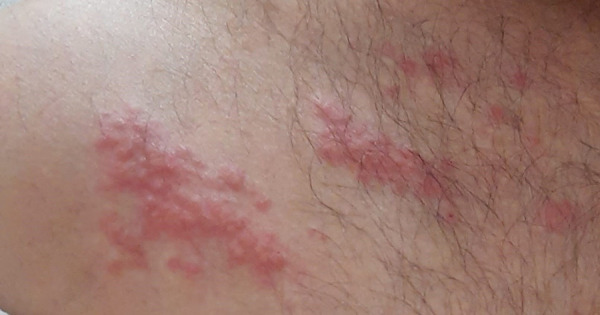
Eythematous vesicular lesions localized along right C5 dermatome.

A 43-year-old male patient diagnosed with asthma, allergic rhinitis, and nasal polyps was receiving inhaled beclomethasone-formoterol, oral montelukast, and nasal fluticasone treatment. The patient was found to have *Penicillium* sensitivity. He had previously been treated with omalizumab for 2 years and with mepolizumab for 5 months due to severe persistent allergic and eosinophilic asthma. Since adequate asthma control could not be achieved, the patient was started on benralizumab treatment. Two days after the first benralizumab injection, the patient presented with localized, blistering, and painful rash on the anterior chest wall. Physical examination revealed erythematous vesicular lesions localized along right C5 dermatome (Figure 1). The patient was evaluated in the dermatology clinic and was diagnosed with HZ and started on oral valacyclovir and topical acyclovir and hydrocortisone treatment. The patient showed significant clinical improvement, and the lesions regressed within 4 weeks. 

The patient was not receiving regular immunosuppressive treatment that could have caused herpetic infection. However, 10 days before the first dose of benralizumab, he had received a 7-day methylprednisolone treatment at a dose of 32 mg/day for an asthma exacerbation. Although it was uncertain whether HZ was related to benralizumab injection, considering that the exact etiology of the eruption could not be determined, we decided to discontinue benralizumab treatment and shared this decision with the patient. However, the patient expressed his desire to continue treatment due to the lack of alternative treatment options for severe persistent eosinophilic asthma. After the opinion and approval of the dermatology department were obtained, benralizumab treatment was continued and the patient has currently received the third dose injection without experiencing recurrent herpetic eruption or any other clinical problem. 

## Discussion 

Recently, many new immunotherapeutic antibodies have been used for asthma. With increasing reports of drug-related side effects, real-life data addressing the drug-induced side effect profile are needed. The present case report is important in this regard. 

Benralizumab and mepolizumab are monoclonal antibodies that both target IL-5. While mepolizumab blocks IL-5 directly, benralizumab acts on the IL-5 receptor alpha chain. Clinical trials with mepolizumab have reported some cases of HZ in patients medicated with the drug, whereas no cases were reported in the placebo arm [3, 4]. Therefore, the recommendation for vaccination against HZ is included in the mepolizumab product label [5]. Although cases of HZ were also detected in clinical trials with benralizumab, the incidence was similar to that in the placebo group [6]. Accordingly, the benralizumab drug information leaflet does not contain any HZ warnings [7]. In 2019, Mishra et al. [8] were the first to publish a case of HZ that may be associated with benralizumab. They noted that this report could raise the possibility of a class effect for the anti-IL-5 monoclonal antibody, as previously only data on mepolizumab were available, but no such data were available for benralizumab [8]. 

A descriptive analysis from the Spanish pharmacovigilance database concerning the safety profile of biologic drugs for severe uncontrolled asthma revealed a statistically significantly increased risk of herpes virus infections in patients taking omalizumab and mepolizumab. In April 2024, the European Medicines Agency added HZ to the list of side effects for mepolizumab [9]. Additionally, analysis of World Health Organization pharmacovigilance data reported an increased risk of HZ in patients taking omalizumab, mepolizumab, and benralizumab [10]. Eosinophils play a role in the immune response to certain viral infections and have antiviral activity [6]. Therefore, the decrease in eosinophils caused by benralizumab may contribute to the susceptibility to HZ. However, it is not possible to say that the herpetic eruption observed in the patient in the presented case is directly related to benralizumab treatment. The development of HZ in the patient may be coincidental. The systemic steroid treatment the patient received before benralizumab treatment may also have predisposed the patient to HZ. In addition, asthma itself is known to increase the susceptibility to HZ [11]. 

Our knowledge about biologics, which are rapidly and newly introduced to our clinical practice in the treatment of asthma, is increasing day by day. Patient data on this subject are valuable in predicting the side effects of treatments and determining the management of biologic treatments in special cases. 

## Authors’ contributions 

Kurtuluş Aksu: Clinical follow-up of the case, writing of the case report, literature research, approval of the final version of the article, and submitting it to the journal. 

Onur Telli: Clinical follow-up of the case, writing of the case report, literature research, approval of the final version of the article, and submitting it to the journal. 

Melis Yağdıran: Clinical follow-up of the case, writing of the case report, literature research, approval of the final version of the article, and submitting it to the journal. 

Fatma Dindar Çelik: Clinical follow-up of the case, writing of the case report, literature research, approval of the final version of the article, and submitting it to the journal. 

Hatice Çelik Tuğlu: Clinical follow-up of the case, writing of the case report, literature research, approval of the final version of the article, and submitting it to the journal. 

Özge Göktürk: Clinical follow-up of the case, writing of the case report, literature research, approval of the final version of the article, and submitting it to the journal. 

## Informed consent 

Consent was obtained from the patient to publish this case report. 

## Funding 

No funding was received for the study. 

## Conflict of interest 

All authors declare that no conflict of interest may have influenced either the conduct or the presentation of the research.
